# Bone Disease in Cystic Fibrosis: Insights into Etiopathogenesis and Advances in Treatment Management

**DOI:** 10.3390/jcm14165657

**Published:** 2025-08-10

**Authors:** Paola Giordano, Giovanna Linguiti, Giuseppina Leonetti, Rosa Maria Pia Casolino, Vanja Granberg, Maria Felicia Faienza

**Affiliations:** 1 Pediatric Unit “B. Trambusti”, Cystic Fibrosis Regional Reference Center, Department of Interdisciplinary Medicine, University of Bari “Aldo Moro”, Piazza G. Cesare 11, 70124 Bari, Italy; paola.giordano@uniba.it (P.G.); susy.leonetti@virgilio.it (G.L.); rosacasolino@gmail.com (R.M.P.C.); 2 Pediatric Unit, Department of Precision and Regenerative Medicine and Ionian Area, University of Bari “Aldo Moro”, Piazza G. Cesare 11, 70124 Bari, Italy; mariafelicia.faienza@uniba.it

**Keywords:** cystic fibrosis, bone disease, cystic fibrosis-related bone disease, bone health, osteopenia, inflammation

## Abstract

Cystic fibrosis (CF) is a multisystemic genetic disorder caused by dysfunctional CF transmembrane conductance regulator (CFTR) protein, leading to impaired chloride and bicarbonate transport. Advances in care have increased patient lifetime, revealing chronic complications such as CF-related bone disease (CFBD), characterized by low bone mineral density and increased fracture risk. CFBD results from a complex interplay of factors including chronic inflammation, nutritional deficiencies, hormonal imbalances, and impaired glucose metabolism. Pro-inflammatory cytokines (e.g., TNF-α, IL-1β, IL-6, and IL-8) promote osteoclastogenesis, disrupting bone remodeling via the RANK/RANKL/OPG pathway. In vivo murine and in vitro studies have elucidated the pathogenic mechanisms underlying CFBD, highlighting CFTR’s role in bone cell function. Diagnosis is based on clinical evaluation, bone densitometry, and laboratory assessments of bone metabolism markers. In this narrative review we highlight the recent scientific evidence on the etiopathogenesis and the current strategies for management of CFBD.

## 1. Introduction

Cystic fibrosis (CF) is a genetic recessive disorder with a multisystemic phenotype caused by dysfunctional CF transmembrane conductance regulator (CFTR) protein, an ion channel responsible for transcellular passage of chloride and bicarbonate, which regulates epithelial surface fluid and mucous viscosity [1]. Decreased CFTR quantity or function leads to the disruption of multiple organ functions, resulting in lung disease, gastrointestinal abnormalities, impaired growth, diabetes, infertility, and other clinical complications. It is estimated that 105,000 people belonging to various ethnicities in 94 countries have been diagnosed with CF [2]. While progressive lung disease characterized by chronic inflammation has long been thought to be the major cause of morbidity in CF, inflammatory manifestations have also been observed in other tissues and organs [3].

**Figure 1:**
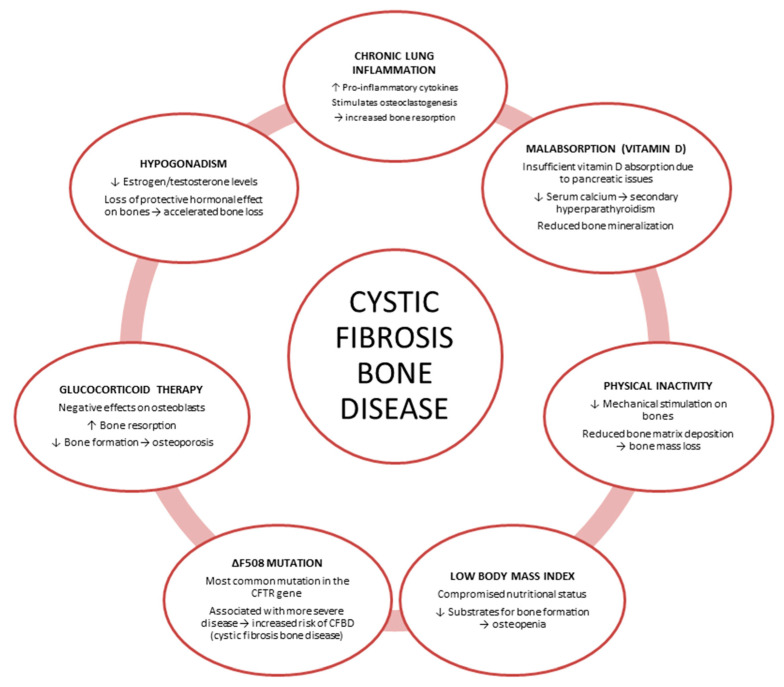
Etiopathogenetic mechanisms of cystic fibrosis bone disease. Cystic fibrosis transmembrane conductance regulator (CFTR); cystic fibrosis bone disease (CFBD).

Over the last few decades, the establishment of specialized reference centers for the treatment and management of patients with CF, the implementation of newborn screening programs, and the availability of protocols to treat and eradicate chronic infections have increased life expectancy to 50 years [4]. Getting older with CF is associated with the development of chronic complications such as CF-related bone disease (CFBD) occurring in around 20% of adolescents and between 55% and 65% of subjects aged 45 years or older [5,6]. CFBD manifests as low bone mineral density (BMD) and severe bone mass reduction, increasing the risk for low trauma fractures, with harmful effects on quality of life and longevity of CF patients [7]. The highest morbidity of ribs and vertebrae fractures in CF results from pain related to cough, ineffective sputum clearance, and pulmonary exacerbation. The causes of impaired bone health status in CF patients are multifactorial (Figure 1). Indeed, the genetically determined CFTR malfunction coupled with poor nutritional status, recurring infections and pro-inflammatory status, altered hormonal status, steroid therapy, physical inactivity, and low levels of vitamin D may all contribute to impaired bone remodeling with consequent decreased bone formation and increased bone resorption [8,9,10].

Vitamin D deficiency in CF patients is associated with increased frequency of pulmonary exacerbations, whereas vitamin D supplementation appears to accelerate recovery from such events. This may be due to vitamin D-induced expression of cathelicidin (LL-37), an antimicrobial peptide whose mRNA levels increase in peripheral blood monocytes. Cathelicidin plays a vital role in preventing *Pseudomonas aeruginosa* colonization in the airways. Moreover, vitamin D can suppress pro-inflammatory cytokine production by airway macrophages, highlighting its immunomodulatory function in the respiratory tract [11]. Bone remodeling is a finely tuned process, regulated by crucial pathways for the balance between bone resorption by osteoclasts and bone formation by osteoblasts [12]. The Receptor activator of nuclear factor κ B (NF-kB) (RANK) and its soluble ligand (sRANKL) regulate osteoclast activity. RANKL promotes the differentiation and fusion of osteoclasts precursors (OCPs) and activates mature osteoclasts towards bone resorption by binding to its specific RANK receptor. Osteoprotegerin (OPG) is a soluble decoy receptor for RANKL which inhibits RANK-RANKL interactions, thereby suppressing osteoclastogenesis and bone resorption. RANKL is a member of the TNF superfamily of ligands. It is produced by both immune cells and bone cells such as osteoblast and osteocytes. RANKL interacts with its receptor RANK on the osteoclast surface, resulting in the activation of the signaling cascade and ultimately inducing the activation of transcriptional factors of osteoclastogenesis. This pathway promotes the maturation, differentiation, and survival of osteoclasts [13]. RANKL expression is significantly regulated by inflammatory signals, particularly in the context of bone-immune interactions. Inflammatory cytokines, such as Tumor Necrosis Factor-alpha (TNF-α), Interleukin-1 (IL-1), Interleukin-6 (IL-6), and Interleukin-17 (IL-17), can induce RANKL expression in numerous cell types, including osteoblasts, osteocytes, and immune cells. This overexpression of RANKL contributes to the formation and activation of osteoclasts, leading to bone resorption, a hallmark of inflammatory bone diseases [14]. On the other hand, the Wnt signaling pathway increases bone mass through several mechanisms, including stem cell renewal, stimulation of pre-osteoblastic replication, induction of osteoblastogenesis, osteoblast inhibition, and osteocyte apoptosis [15]. The activity of the canonical Wnt signaling pathway in bone cells generally leads to the formation of bone tissue and the maintenance of normal bone density in adults [16]. Several acquired and congenital pediatric diseases showed an imbalance in the RANK/RANKL/OPG axis, which causes an impairment of bone health [17,18,19].

This narrative review aims to summarize the most recent scientific evidence on the etiopathogenesis of CFBD, by presenting recent in vivo and in vitro studies, and to discuss current therapeutic strategies.

## 2. Roles of Inflammatory Cytokines in CFBD

Patients with CF typically experience chronic inflammation, primarily driven by persistent pulmonary infection, epithelial dysfunction, and dysregulation of both innate and adaptive immune responses. In CF lungs, there is a significant accumulation of alveolar macrophages and neutrophils. The ongoing infection results in continuous production of pro-inflammatory cytokines such as TNF-α, IL-1β, and IL-6. This sustained inflammatory environment plays a key role in the disruption of bone homeostasis [20]. Cytokines function by binding to specific receptors on the surface of target cells, triggering intracellular signaling pathways that alter gene expression and cellular activity. The previously mentioned cytokines play a significant role in bone remodeling by promoting osteoclastogenesis and inhibiting osteoblast function [3]. TNF-α is produced by activated macrophages, T cells, B cells, and natural killer cells. It directly binds to its receptor on the cell surface and synergizes with RANKL signaling to promote osteoclastogenesis. TNF-α also induces the production of Dickkopf-1 (DKK1), a critical inhibitor of the Wnt pathway, suppressing bone formation and thus contributing to inflammatory bone loss. Therefore, TNF-α induces bone resorption by both increasing osteoclastic activity and decreasing osteoblastic bone formation in many inflammatory diseases. These mechanisms of action also have therapeutic implications, as using inhibitory antibodies such as infliximab could improve bone health in inflammatory diseases [21]. IL-1β is produced by monocytes and macrophages. RANKL expression on stromal cells can be induced by IL-1β, which directly links to the activation of osteoclast precursors. IL-6 plays a multifaceted role during bone remodeling. IL-6 is produced by monocytes and macrophages. It regulates RANK/RANKL signaling, increasing RANKL expression, but it has an ambiguous role because it also suppresses the osteoclast precursor differentiation via NF-kB signaling. Additionally, high levels of IL-8, another pro-inflammatory cytokine that recruits and activates neutrophils, thereby sustaining the inflammatory response, have been detected in CF patients. IL-8 indirectly influences bone remodeling by promoting the expression of RANKL, thus enhancing osteoclastogenesis. IL-8 may also impair osteoblast function, further disrupting the balance between bone formation and resorption. Conversely, during chronic lung infections, reduced levels of IL-10, an anti-inflammatory cytokine known to support osteoblast function and survival, have been observed [3].

## 3. Impaired Glucose Metabolism in CF

The chronic inflammatory state in CF affects bone remodeling, both directly and indirectly, by influencing glucose metabolism. Indeed, pro-inflammatory cytokines induce insulin resistance, further impairing glucose metabolism and exacerbating bone loss. Cystic fibrosis-related diabetes (CFRD) usually appears around the second decade of life due to insulin deficiency. Insulin is anabolic for bone, by promoting osteoblast activity and bone formation. CFRD, which combines features of both insulin deficiency and resistance, results from progressive pancreatic destruction caused by thick secretions and fibrosis. This metabolic disturbance extends beyond glucose regulation, intersecting with bone health through complex signaling pathways. Moreover, the hyperglycemia characteristic of CFRD exacerbates skeletal complications, as elevated blood glucose levels increase the production of advanced glycation end-products (AGEs), which impair osteoblast function and enhance osteoclast-mediated resorption. Additionally, AGEs disrupt collagen cross-linking, reducing bone quality and increasing fragility [3]. This bidirectional relationship underscores the complexity of managing bone health in CF patients. The bone–pancreas loop underscores the close interconnection between skeletal and glucose metabolism. Bone-derived factors such as osteocalcin influence insulin secretion and sensitivity, thereby contributing to systemic glucose homeostasis. Conversely, conditions that impair glucose regulation can negatively impact bone remodeling processes. This interplay is particularly relevant in CF, where chronic inflammation, malnutrition, and corticosteroid therapy contribute to both metabolic and skeletal deterioration [22]. In individuals with type 1 diabetes, which shares pathophysiological features with CFRD, studies demonstrate elevated serum levels of sclerostin and DKK-1, both inhibitors of the Wnt signaling pathway essential for bone formation. These findings are relevant for CF patients, as similar mechanisms likely contribute to skeletal fragility by inhibiting osteoblast activity, reducing bone formation, and weakening bone integrity [23].

## 4. Current State of Knowledge on CFBD: In Vitro, In Vivo, and Ex Vivo Studies

In vitro, in vivo, and ex vivo studies have facilitated a more comprehensive elucidation of the underlying mechanisms involved in the CFBD pathogenesis (Table 1).

### 4.1. In Vitro Studies

Tissue sections from young mouse jaws and fetal human jaws have shown that CFTR is expressed by osteoblasts, odontoblasts, chondrocytes, and myocytes, with mutations directly impairing the function of these cell types [24]. Le Henaff et al. identified the molecular mechanism by which the ΔF508-CFTR mutation negatively affects osteoblastic function and differentiation. These mechanisms have been characterized primarily in murine models, and their applicability to human bone physiology remains to be fully elucidated. Specifically, this mutation leads to reduced osteoblast gene expression and osteogenic activity, as a consequence of NF-κB hyperactivity and suppressed Wnt/β-catenin signaling, while cell proliferation remains unaffected. Notably, the mutation impairs osteoblast differentiation and function ex vivo, demonstrating that the dysfunction occurs independently of nutritional, inflammatory, or hormonal status [25]. Dumortier et al. observed a delay in osteoblast lineage commitment accompanied by dysregulated expression of key molecular markers, including an elevated RANKL/OPG ratio and reduced Bone Morphogenetic Protein 2 (BMP2) levels, indicating that CFTR mutations may disrupt bone homeostasis by altering the balance between bone formation and resorption [26].

### 4.2. In Vivo Studies

Genetically modified mouse models with specific alterations in CFTR have been developed to study the pathophysiology of cystic fibrosis [37].

Cftr^−^/^−^ mice have shown that deletion of the gene leads to low bone density and alterations in bone microarchitecture. The first study involving Cftr^−^/^−^ knockout mice reported that the mutant mice were smaller than controls and suffered from severe osteopenia affecting both trabecular and cortical bone. This phenotype was linked to a marked reduction in new bone formation and increased bone resorption, suggesting that osteopenia results from an imbalance in bone remodeling. Importantly, measurements were performed at 3 weeks of age, as most of the mice died from intestinal obstruction around that time [27]. Similar results were obtained by Haston et al. [28] and Paradis et al. [29], who used microCT imaging to demonstrate that CF adult mice expressing either reduced or wild-type levels of ΔF508-CFTR exhibit osteopenia.

Le Henaff et al. further demonstrated that the F508del mutation leads to reduced bone mass and bone formation regardless of sex. Their data suggest that while the mutation does not increase the number of osteoclasts, it alters their function. Specifically, the F508del mutation dissociates osteoblastic and osteoclastic activity, increasing bone resorption during the trabecular bone development phase [30]. Pashuck et al. identified sex-specific differences in bone metabolism among CF mice. They observed increased bone formation in male CF mice compared to females, a finding that may align with clinical differences in CF presentation between sexes [31]. However, although male mice show increased bone formation, this is paralleled by increased resorption, ultimately still resulting in reduced bone density [31]. This study employed Cftr^−^/^−^ mice with corrected intestinal CFTR deficiency via a transgene expressing human CFTR, thereby avoiding the nutritional complications seen in earlier models. In the study by Dif et al. [27], Cftr^−^/^−^ mice died from intestinal obstruction, typically around three weeks of age, thereby requiring all analyses to be conducted within this time frame. Stalvey et al. demonstrated that CFTR is expressed in osteoblasts and that its inactivation results in impaired osteoblast differentiation, delayed bone formation, and disrupted canonical Wnt signaling. Although CFTR is not expressed by osteoclasts, the observed increase in osteoclastogenesis is likely secondary to reduced OPG expression [32]. In a related study, Le Henaff et al. found that in a mouse model of CF, osteoblasts express the intermediate filament protein keratin 8 (Krt8), and that deletion of Krt8 in wild-type mice causes osteopenia due to impaired bone formation. The deletion downregulates osteoblastic markers such as Runx2 and Col1a1, indicating a critical role of Krt8 in osteoblast differentiation. Strikingly, Krt8 deletion in F508del-Cftr mice rescued osteoblast differentiation and osteogenic activity, attenuating bone loss. These findings may represent a promising therapeutic avenue to target impaired bone formation in CF patients [33].

### 4.3. Ex Vivo Studies

King et al. [38] have shown that the ΔF508 mutation is in itself an independent risk factor for FCBD. A study examining human bone sections confirmed that CFTR is expressed by osteoblasts, further supporting a direct role of CFTR in human bone metabolism [34].

Further support for a direct role of CFTR in musculoskeletal function comes from studies in Cftr^−^/^−^ mouse models and human muscle biopsies, which demonstrated that the absence of CFTR in skeletal muscle leads to muscle dysfunction. This is characterized by impaired calcium homeostasis, upregulation of inflammatory genes, and diaphragmatic weakness during pulmonary infections. These findings reveal a previously unrecognized role of CFTR in skeletal muscle physiology, with potential implications for cachexia and respiratory muscle failure in CF patients [35]. A study by Le Heron et al. found that in bone cells from non-classic CF patients, loss of CFTR activity promotes inflammation-driven bone resorption via both reduced OPG production and increased prostaglandin E2 (PGE2) release. This suggests that CFTR deficiency contributes to osteopenia independently of nutritional status or pulmonary disease severity [36].

## 5. Current and Future Therapeutic Interventions: Nutrition/Physical Activity/Pharmacological Treatments

The management of bone disease in CF currently relies on a comprehensive, multidisciplinary approach that includes early monitoring through DXA, optimization of nutritional status, supplementation with vitamin D, calcium, vitamin K, and other essential micronutrients, promotion of physical activity, and, in selected cases, the use of anti-osteoporotic agents such as bisphosphonates. A critical area of development involves early biomarkers of bone disease. Current diagnosis of CFBD is largely based on DXA. The use of serum biomarkers such as CTX (C-terminal telopeptide), P1NP (procollagen type I N-terminal propeptide) [39], and osteocalcin could enable earlier and more dynamic assessment of bone turnover. Advanced imaging techniques (such as Trabecular Bone Score (TBS) [40] or high-resolution MRI) are also gaining traction as tools to assess bone quality beyond density, potentially allowing for earlier and more targeted interventions before the onset of fractures or skeletal deformities.

### 5.1. Nutrition

From a nutritional standpoint, one of the primary goals is to maintain adequate serum levels of vitamin D. The Cystic Fibrosis Foundation’s (CFF) and the Endocrine Society’s guidelines recommend a serum vitamin D concentration of at least 30 ng/mL [41]. Vitamin D levels are insufficient (20–30 ng/mL) in 36% of CF pediatric subjects and 63% adults; instead, the prevalence of vitamin levels insufficiency (below 20 ng/mL) is 27% of pediatric/adolescent and 35% of adult CF patients, according to the meta-analysis of Farahbakhsh et al. [42]. To reach this target, daily supplementation with cholecalciferol or ergocalciferol is often necessary [41,43]. In CF patients, clinical guidelines often recommend achieving serum 25(OH)D levels above 30 ng/mL, and in some cases > 40 ng/mL. These targets are higher than those for the general population, aiming to compensate for chronic malabsorption and to better support bone and immune health. In contrast, in the general population, serum levels > 20 ng/mL are considered adequate according to many guidelines [44]. Calcium intake should be primarily dietary, with recommended daily amounts of ±1000 mg [45], and supplemented as needed. Vitamin K plays a pivotal role in bone metabolism, particularly in promoting mineralization. In CF patients with pancreatic insufficiency, vitamin K deficiency is common; hence, daily supplementation (1–10 mg/d) is often indicated, depending on plasma levels and pancreatic function [43]. Magnesium [46] and phosphorus [47] are also crucial micronutrients for bone health and should be regularly monitored and corrected if imbalances are detected. Moreover, endocrine dysfunctions (such as hypogonadism, which impairs bone formation, or diabetes [48], which increases skeletal fragility) must be identified and treated appropriately.

### 5.2. Physical Activity

A cornerstone of both prevention and treatment of CFBD is regular physical activity [49], which serves as a highly effective intervention to counteract bone loss, enhance overall patient function, and support the efficacy of nutritional therapies. The benefits of physical activity are multifaceted: it stimulates bone formation through mechanical loading, which enhances osteoblast activity and suppresses osteoclast function, thus preserving skeletal architecture and strength [50]. It also increases muscle mass, an essential factor for skeletal support, posture, and daily mobility. Furthermore, physical exercise improves balance and coordination, significantly reducing the risk of falls and fractures, particularly in patients with osteopenia or osteoporosis. Notably, regular exercise enhances intestinal calcium absorption, supports the regulation of anabolic hormones such as IGF-1 and growth hormone [51], and contributes to the reduction in systemic inflammation (a known exacerbator of bone loss). In practical terms, the exercise program should be individualized, continuous, and tailored to the patient’s clinical condition.

### 5.3. Pharmacological Treatments

In selected cases, pharmacological treatment may be indicated under close specialist supervision. These include patients with markedly reduced bone mineral density (Z-score < −2 SD in adolescents or T-score < −2.5 in adults), particularly when associated with vertebral, rib, or other low-trauma fractures; chronic and disabling bone pain; rapid deterioration of bone density as documented by DXA; or the presence of additional risk factors such as prolonged corticosteroid therapy, hypogonadism, persistent malnutrition, or CFRD. Among the most established therapeutic options are bisphosphonates, antiresorptive drugs that inhibit osteoclast activity, slow pathological bone remodeling, and help stabilize or improve bone density [49,52]. Oral bisphosphonates such as alendronate (70 mg weekly) and risedronate (35 mg weekly) are commonly prescribed [53]. However, in many CF patients, gastrointestinal absorption is compromised due to pancreatic insufficiency or accelerated intestinal transit. Consequently, intravenous bisphosphonates, including zoledronate (5 mg annually) or ibandronate (every three months), are often preferred in patients with poor absorption, low adherence, or more severe disease phenotypes [53]. Despite their efficacy, bisphosphonates are not without side effects. Oral formulations are frequently associated with gastrointestinal disturbances (nausea, reflux, esophagitis), while intravenous administration (particularly with zoledronate) may cause flu-like symptoms after the first dose. Transient hypocalcemia can also occur, especially in patients with uncorrected vitamin deficiencies. One of the most feared complications is osteonecrosis of the jaw (ONJ), a serious condition characterized by persistent pain, exposed bone, infection, and impaired mastication. Another pharmacological option gaining attention in CFBD is denosumab, a fully human monoclonal antibody that binds to RANKL, directly inhibiting osteoclast formation and activity. Denosumab offers theoretical advantages in CF patients: it is not renally excreted, does not accumulate in bone, and exerts a systemic and reversible effect [54], when administered subcutaneously every six months (60 mg). It may be a viable alternative for patients who cannot tolerate bisphosphonates, have impaired renal function, or are unable to take oral medications (a common scenario in advanced pancreatic insufficiency). However, the use of denosumab in CF remains off-label, as current evidence is limited to animal experiments [38], case reports, and small studies. It is important to note, however, that abrupt discontinuation of denosumab can lead to a rebound in bone resorption, with rapid loss of previously gained BMD and, in rare cases, multiple vertebral compression fractures. As such, bisphosphonate “bridging” therapy is recommended upon discontinuation to prevent rebound effects [55]. Recent advances in CFBD research are paving the way for innovative therapeutic strategies aimed at overcoming the limitations of conventional treatments. Among these, novel osteoactive agents, advanced biomarkers, and personalized medicine approaches are garnering increasing interest and hold promise for revolutionizing skeletal care in this population.

A particularly promising area is the development of sclerostin inhibitors, such as romosozumab, recently approved for postmenopausal osteoporosis at high fracture risk [56]. Romosozumab exerts a dual action: it strongly stimulates bone formation via osteoblast activation and simultaneously reduces osteoclastic resorption by inhibiting sclerostin (a protein produced by osteocytes that physiologically suppresses the Wnt signaling pathway, essential for bone anabolism). Results in the general population are encouraging, with rapid and significant gains in bone density, particularly at the spine. However, in CF patients, romosozumab remains investigational; robust data on its safety, efficacy, and interactions with the unique inflammatory and metabolic profile of CF are lacking. Moreover, rare cardiovascular events reported in clinical trials restrict its use to patients without recent ischemic heart disease [53]. Consequently, this treatment must be used cautiously in patients with heart failure and comorbidities because of the associated cardiovascular risk. In parallel, research is exploring alternative anabolic peptides beyond teriparatide, aiming to selectively enhance osteoblastic activity [57]. These include analogs of parathyroid hormone (PTH) or synthetic peptides that mimic the actions of bone growth factors, designed to stimulate bone formation without increasing resorption. Though still in experimental stages for CF, preclinical findings are promising, especially in younger patients with severe bone demineralization unresponsive to standard therapies.

## 6. Conclusions

Ongoing advancements in the clinical management of CF have markedly extended patient life expectancy. While this progress represents a major milestone, it has also highlighted the growing burden of long-term comorbidities associated with CF, including bone disease and metabolic conditions such as diabetes. Within this evolving clinical context, a comprehensive and individualized approach has become essential to anticipate and effectively address these complications, with the overarching aim of promoting sustained quality of life and preserving patients’ functional autonomy. Consequently, the focus of CFBD research is shifting toward increasingly precise, proactive, and individualized strategies, aiming not only to preserve bone health but also to support the long-term well-being and independence of individuals living with CF.

## Figures and Tables

**Table 1:** Summary of cited studies: author, model used, and key findings.

Author and Year	Model Used	Outcomes
Bronckers et al., 2010 [24]	Animals and human tissues	CFTR dysfunction may contribute to osteopenia in both CF patients and CFTR-deficient mice due to its role on bone cells.
Le Henaff et al., 2015 [25]	Mouse cell line	The prevalent ΔF508-CFTR mutation impairs osteoblast differentiation and function by increasing NF-κB signaling and reducing Wnt/β-catenin signaling.
Dumortier et al., 2025 [26]	Human cell line	CFTR mutation delays osteoblast differentiation and regeneration.
Dif et al., 2004 [27]	Mouse model	CFTR mutation is directly linked to osteopenia.
Haston et al., 2008 [28]	Mouse model	CFTR loss causes persistent osteopenia and bone abnormalities, independent of sex or haplotype.
Paradis et al., 2010 [29]	Mouse model	ΔF508-CFTR expression causes osteopenia, indicating CFTR dysfunction directly contributes to CF bone disease.
Le Henaff et al., 2012 [30]	Mouse model	F508del-CFTR reduces bone formation and causes osteopenia and bone architecture defects.
Pashuck et al., 2009 [31]	Mouse model	Reduced bone formation in female CF mice, increased in males.
Stalvey et al., 2013 [32]	Mouse model	CFTR inactivation impairs osteoblast function, increases osteoclastogenesis, and disrupts Wnt signaling, contributing to CF-related bone disease.
Le Henaff et al., 2016 [33]	Mouse model	Krt8 targeting restores bone formation and reduces osteopenia in F508del-CFTR mice.
Shead et al., 2007 [34]	Human tissue and cell line	CFTR is expressed in human osteoblasts, osteocytes, and osteoclasts
Divangahi et al., 2009 [35]	Human and mouse tissues and cell line	Unrecognized role of CFTR in skeletal muscle linked to cachexia and respiratory dysfunction in CF.
Le Heron et al., 2010 [36]	Human cell line	CFTR loss may increase inflammation-driven bone resorption, contributing to early bone loss in CF children.

Cystic fibrosis transmembrane conductance regulator (CFTR); Nuclear Factor kB (NF-kB); cystic fibrosis (CF).

## Data Availability

No new data were created or analyzed in this study. Data sharing is not applicable to this article.

## Abbreviations

The following abbreviations are used in this manuscript:

CF	Cystic fibrosis
CFTR	CF transmembrane conductance regulator
CFBD	CF-related bone disease
BMD	Bone mineral density
CFRD	CF-related diabetes

## References

[1] Ong T., Ramsey B.W. (2015). Update in Cystic Fibrosis 2014. Am. J. Respir. Crit. Care Med..

[2] Fuhrer M., Zampoli M., Abriel H. (2024). Diagnosing Cystic Fibrosis in Low- and Middle-Income Countries: Challenges and Strategies. Orphanet J. Rare Dis..

[3] Fonseca Ó., Gomes M.S., Amorim M.A., Gomes A.C. (2023). Cystic Fibrosis Bone Disease: The Interplay between CFTR Dysfunction and Chronic Inflammation. Biomolecules.

[4] Vavrina K., Griffin T.B., Jones A.M., Schindler T., Bui T.N., Sankararaman S. (2025). Evolving Nutrition Therapy in Cystic Fibrosis: Adapting to the CFTR Modulator Era. Nutr. Clin. Pract..

[5] Mora Vallellano J., Delgado Pecellín C., Delgado Pecellín I., Quintana Gallego E., López-Campos J.L. (2021). Evaluation of Bone Metabolism in Children with Cystic Fibrosis. Bone.

[6] Jad R., Ma X., Stanojevic S., Illango A., Tullis E., Gilmour J., Goss C.H., Strug L.J., Stephenson A.L. (2024). Longitudinal Changes in BMD in Adults with Cystic Fibrosis. J. Bone Miner. Res..

[7] Cobb C., Wu M., Tangpricha V. (2025). Cystic Fibrosis-Related Bone Disease: An Update on Screening, Diagnosis, and Treatment. Ther. Adv. Endocrinol. Metab..

[8] Conway S.P. (2000). Osteoporosis and Osteopenia in Adults and Adolescents with Cystic Fibrosis: Prevalence and Associated Factors. Thorax.

[9] Ionescu A.A., Nixon L.S., Evans W.D., Stone M.D., Lewis-Jenkins V., Chatham K., Shale D.J. (2000). Bone Density, Body Composition, and Inflammatory Status in Cystic Fibrosis. Am. J. Respir. Crit. Care Med..

[10] Putman M.S., Anabtawi A., Le T., Tangpricha V., Sermet-Gaudelus I. (2019). Cystic Fibrosis Bone Disease Treatment: Current Knowledge and Future Directions. J. Cyst. Fibros..

[11] Fenercioglu A.K. (2024). The Anti-Inflammatory Roles of Vitamin D for Improving Human Health. Curr. Issues Mol. Biol..

[12] Boyce B.F., Xing L. (2008). Functions of RANKL/RANK/OPG in Bone Modeling and Remodeling. Arch. Biochem. Biophys..

[13] Zhou P., Zheng T., Zhao B. (2022). Cytokine-Mediated Immunomodulation of Osteoclastogenesis. Bone.

[14] Kitaura H., Marahleh A., Ohori F., Noguchi T., Shen W.R., Qi J., Nara Y., Pramusita A., Kinjo R., Mizoguchi I. (2020). Osteocyte-Related Cytokines Regulate Osteoclast Formation and Bone Resorption. Int. J. Mol. Sci..

[15] Houschyar K.S., Tapking C., Borrelli M.R., Popp D., Duscher D., Maan Z.N., Chelliah M.P., Li J., Harati K., Wallner C. (2019). Wnt Pathway in Bone Repair and Regeneration—What Do We Know So Far. Front. Cell Dev. Biol..

[16] Holmen S.L., Zylstra C.R., Mukherjee A., Sigler R.E., Faugere M.-C., Bouxsein M.L., Deng L., Clemens T.L., Williams B.O. (2005). Essential Role of β-Catenin in Postnatal Bone Acquisition. J. Biol. Chem..

[17] Brunetti G., D’Amato G., Chiarito M., Tullo A., Colaianni G., Colucci S., Grano M., Faienza M.F. (2019). An Update on the Role of RANKL–RANK/Osteoprotegerin and WNT-ß-Catenin Signaling Pathways in Pediatric Diseases. World J. Pediatr..

[18] Faienza M.F., Ventura A., Colucci S., Cavallo L., Grano M., Brunetti G. (2016). Bone Fragility in Turner Syndrome: Mechanisms and Prevention Strategies. Front. Endocrinol..

[19] Faienza M.F., Brunetti G., Ventura A., Piacente L., Messina M.F., De Luca F., Ciccarelli M., Oranger A., Mori G., Natale M.P. (2015). Mechanisms of Enhanced Osteoclastogenesis in Girls and Young Women with Turner’s Syndrome. Bone.

[20] Roesch E.A., Nichols D.P., Chmiel J.F. (2018). Inflammation in Cystic Fibrosis: An Update. Pediatr. Pulmonol..

[21] Ryan B.M., Russel M.G.V.M., Schurgers L., Wichers M., Sijbrandij J., Stockbrugger R.W., Schoon E. (2004). Effect of Antitumour Necrosis Factor-α Therapy on Bone Turnover in Patients with Active Crohn’s Disease: A Prospective Study. Aliment. Pharmacol. Ther..

[22] Faienza M.F., Luce V., Ventura A., Colaianni G., Colucci S., Cavallo L., Grano M., Brunetti G. (2015). Skeleton and Glucose Metabolism: A Bone-Pancreas Loop. Int. J. Endocrinol..

[23] Brunetti G., D’Amato G., De Santis S., Grano M., Faienza M.F. (2021). Mechanisms of Altered Bone Remodeling in Children with Type 1 Diabetes. World J. Diabetes.

[24] Bronckers A., Kalogeraki L., Jorna H.J.N., Wilke M., Bervoets T.J., Lyaruu D.M., Zandieh-Doulabi B., DenBesten P., De Jonge H. (2010). The Cystic Fibrosis Transmembrane Conductance Regulator (CFTR) Is Expressed in Maturation Stage Ameloblasts, Odontoblasts and Bone Cells. Bone.

[25] Le Henaff C., Mansouri R., Modrowski D., Zarka M., Geoffroy V., Marty C., Tarantino N., Laplantine E., Marie P.J. (2015). Increased NF-κB Activity and Decreased Wnt/β-Catenin Signaling Mediate Reduced Osteoblast Differentiation and Function in ΔF508 Cystic Fibrosis Transmembrane Conductance Regulator (CFTR) Mice. J. Biol. Chem..

[26] Dumortier C., Frauenpreis A., Hoarau A., Ryan A.L., Gangloff S.C., Danopoulos S., Velard F., Al Alam D. (2025). CFTR Mutation Is Associated with Bone Differentiation Abnormalities in Cystic Fibrosis. J. Cyst. Fibros..

[27] Dif F., Marty C., Baudoin C., De Vernejoul M.-C., Levi G. (2004). Severe Osteopenia in CFTR-Null Mice. Bone.

[28] Haston C.K., Li W., Li A., Lafleur M., Henderson J.E. (2008). Persistent Osteopenia in Adult Cystic Fibrosis Transmembrane Conductance Regulator–Deficient Mice. Am. J. Respir. Crit. Care Med..

[29] Paradis J., Wilke M., Haston C.K. (2010). Osteopenia in Cftr-deltaF508 Mice. J. Cyst. Fibros..

[30] Le Henaff C., Gimenez A., Haÿ E., Marty C., Marie P., Jacquot J. (2012). The F508del Mutation in Cystic Fibrosis Transmembrane Conductance Regulator Gene Impacts Bone Formation. Am. J. Pathol..

[31] Pashuck T.D., Franz S.E., Altman M.K., Wasserfall C.H., Atkinson M.A., Wronski T.J., Flotte T.R., Stalvey M.S. (2009). Murine Model for Cystic Fibrosis Bone Disease Demonstrates Osteopenia and Sex-Related Differences in Bone Formation. Pediatr. Res..

[32] Stalvey M.S., Clines K.L., Havasi V., McKibbin C.R., Dunn L.K., Chung W.J., Clines G.A. (2013). Osteoblast CFTR Inactivation Reduces Differentiation and Osteoprotegerin Expression in a Mouse Model of Cystic Fibrosis-Related Bone Disease. PLoS ONE.

[33] Le Henaff C., Faria Da Cunha M., Hatton A., Tondelier D., Marty C., Collet C., Zarka M., Geoffroy V., Zatloukal K., Laplantine E. (2016). Genetic Deletion of Keratin 8 Corrects the Altered Bone Formation and Osteopenia in a Mouse Model of Cystic Fibrosis. Hum. Mol. Genet..

[34] Shead E.F., Haworth C.S., Condliffe A.M., McKeon D.J., Scott M.A., Compston J.E. (2007). Cystic Fibrosis Transmembrane Conductance Regulator (CFTR) Is Expressed in Human Bone. Thorax.

[35] Divangahi M., Balghi H., Danialou G., Comtois A.S., Demoule A., Ernest S., Haston C., Robert R., Hanrahan J.W., Radzioch D. (2009). Lack of CFTR in Skeletal Muscle Predisposes to Muscle Wasting and Diaphragm Muscle Pump Failure in Cystic Fibrosis Mice. PLoS Genet..

[36] Le Heron L., Guillaume C., Velard F., Braux J., Touqui L., Moriceau S., Sermet-Gaudelus I., Laurent-Maquin D., Jacquot J. (2010). Cystic Fibrosis Transmembrane Conductance Regulator (CFTR) Regulates the Production of Osteoprotegerin (OPG) and Prostaglandin (PG) E2 in Human Bone. J. Cyst. Fibros..

[37] Scholte B.J., Davidson D.J., Wilke M., De Jonge H.R. (2004). Animal Models of Cystic Fibrosis. J. Cyst. Fibros..

[38] King S.J., Topliss D.J., Kotsimbos T., Nyulasi I.B., Bailey M., Ebeling P.R., Wilson J.W. (2005). Reduced Bone Density in Cystic Fibrosis: ΔF508 Mutation Is an Independent Risk Factor. Eur. Respir. J..

[39] Szulc P., Naylor K., Pickering M.-E., Hoyle N., Eastell R., Leary E. (2018). Use of CTX-I and PINP as Bone Turnover Markers: National Bone Health Alliance Recommendations to Standardize Sample Handling and Patient Preparation to Reduce Pre-Analytical Variability. Ann. Biol. Clin..

[40] Shevroja E., Lamy O., Kohlmeier L., Koromani F., Rivadeneira F., Hans D. (2017). Use of Trabecular Bone Score (TBS) as a Complementary Approach to Dual-Energy X-Ray Absorptiometry (DXA) for Fracture Risk Assessment in Clinical Practice. J. Clin. Densitom..

[41] Bergagnini-Kolev M.C., Hsu S., Aitken M.L., Goss C.H., Hoofnagle A.N., Zelnick L.R., Lum D., Best C.M., Thummel K.E., Kestenbaum B.R. (2023). Metabolism and Pharmacokinetics of Vitamin D in Patients with Cystic Fibrosis. J. Steroid Biochem. Mol. Biol..

[42] Farahbakhsh N., Fatahi S., Shirvani A., Motaharifard M.S., Mohkam M., Tabatabaii S.A., Khanbabaee G., Yaghoobpoor S., Davoodi S.Z., Hosseini A.H. (2024). Vitamin D Deficiency in Patients with Cystic Fibrosis: A Systematic Review and Meta-Analysis. J. Health Popul. Nutr..

[43] Wilschanski M., Munck A., Carrion E., Cipolli M., Collins S., Colombo C., Declercq D., Hatziagorou E., Hulst J., Kalnins D. (2024). ESPEN-ESPGHAN-ECFS Guideline on Nutrition Care for Cystic Fibrosis. Clin. Nutr..

[44] Bertoldo F., Cianferotti L., Di Monaco M., Falchetti A., Fassio A., Gatti D., Gennari L., Giannini S., Girasole G., Gonnelli S. (2022). Definition, Assessment, and Management of Vitamin D Inadequacy: Suggestions, Recommendations, and Warnings from the Italian Society for Osteoporosis, Mineral Metabolism and Bone Diseases (SIOMMMS). Nutrients.

[45] (2011). Dietary Reference Intakes for Calcium and Vitamin D.

[46] Rondanelli M., Faliva M.A., Tartara A., Gasparri C., Perna S., Infantino V., Riva A., Petrangolini G., Peroni G. (2021). An Update on Magnesium and Bone Health. BioMetals.

[47] Serna J., Bergwitz C. (2020). Importance of Dietary Phosphorus for Bone Metabolism and Healthy Aging. Nutrients.

[48] Kanazawa I., Sugimoto T. (2018). Diabetes Mellitus-Induced Bone Fragility. Intern. Med..

[49] Sermet-Gaudelus I., Bianchi M.L., Garabédian M., Aris R.M., Morton A., Hardin D.S., Elkin S.L., Compston J.E., Conway S.P., Castanet M. (2011). European Cystic Fibrosis Bone Mineralisation Guidelines. J. Cyst. Fibros..

[50] Faienza M.F., Urbano F., Chiarito M., Lassandro G., Giordano P. (2023). Musculoskeletal Health in Children and Adolescents. Front. Pediatr..

[51] Birzniece V. (2019). Exercise and the Growth Hormone–Insulin-like Growth Factor Axis. Curr. Opin. Endocr. Metab. Res..

[52] Conwell L.S., Chang A.B. (2014). Bisphosphonates for Osteoporosis in People with Cystic Fibrosis. Cochrane Database Syst. Rev..

[53] Ullal J., Kutney K., Williams K.M., Weber D.R. (2022). Treatment of Cystic Fibrosis Related Bone Disease. J. Clin. Transl. Endocrinol..

[54] Hildebrand G.K., Patel P., Kasi A. (2025). Denosumab. StatPearls.

[55] Lamy O., Stoll D., Aubry-Rozier B., Rodriguez E.G. (2019). Stopping Denosumab. Curr. Osteoporos. Rep..

[56] Cosman F., Crittenden D.B., Adachi J.D., Binkley N., Czerwinski E., Ferrari S., Hofbauer L.C., Lau E., Lewiecki E.M., Miyauchi A. (2016). Romosozumab Treatment in Postmenopausal Women with Osteoporosis. N. Engl. J. Med..

[57] Black D.M., Greenspan S.L., Ensrud K.E., Palermo L., McGowan J.A., Lang T.F., Garnero P., Bouxsein M.L., Bilezikian J.P., Rosen C.J. (2003). The Effects of Parathyroid Hormone and Alendronate Alone or in Combination in Postmenopausal Osteoporosis. N. Engl. J. Med..

